# NMR-Based Metabolomics Approach to Investigate the Effects of Fruits of *Acanthopanax sessiliflorus* in a High-Fat Diet Induced Mouse Model

**DOI:** 10.3390/metabo11080505

**Published:** 2021-07-31

**Authors:** Bo-Ram Choi, Dahye Yoon, Hyoung-Geun Kim, Seon Min Oh, Yung Choon Yoo, Young-Seob Lee, Kwan-Woo Kim, Tae-Hoo Yi, Dae Young Lee

**Affiliations:** 1 Department of Herbal Crop Research, National Institute of Horticultural and Herbal Science, RDA, Eumseong 27709, Korea; bmcbr@korea.kr (B.-R.C.); dahyeyoon@korea.kr (D.Y.); seonmin88@korea.kr (S.M.O.); youngseoblee@korea.kr (Y.-S.L.); swamp1@korea.kr (K.-W.K.); 2 Graduate School of Biotechnology and Department of Oriental Medicine Biotechnology, Kyung Hee University, Yongin 17104, Korea; zwang05@khu.ac.kr (H.-G.K.); drhoo@khu.ac.kr (T.-H.Y.); 3 Department of Microbiology, College of Medicine, Konyang University, Daejeon 35365, Korea; yc_yoo@konyang.ac.kr

**Keywords:** metabolomics, NMR spectroscopy, *Acanthopanax sessiliflorus* fruits, obesity, high-fat diet

## Abstract

The prevalence of obesity is rapidly increasing and is recognized as a serious health problem. To investigate metabolic changes in an obese model after administration of *Acanthopanax sessiliflorus*, mice were divided into four groups: normal diet, high-fat diet (HFD), HFD with treatment fenofibrate, and *A. sessiliflorus* fruit extract. The liver tissue of mice was analyzed using nuclear magnetic resonance (NMR) spectrometry-based metabolomics. In multivariate statistical analyses, the HFD group was discriminated from the normal diet group, and the group fed *A. sessiliflorus* fruit was discriminated from the HFD group. In biomarker analysis between the HFD group and the group fed *A. sessiliflorus* fruit, alanine, inosine, formate, pyroglutamate, taurine, and tyrosine, with AUC values of 0.7 or more, were found. The levels of these metabolites were distinguished from the HFD mouse model. Changes in these metabolites were confirmed to act on metabolic pathways related to antioxidant activity.

## 1. Introduction

Obesity, defined as having a body mass index (BMI) ≥ 30 kg/m^2^ [1], is a complex and multifactorial syndrome. Genetics, lifestyle (such as dietary habits and physical activity patterns), and their interactions are involved in the prevalence of obesity [2] The prevalence of obesity is increasing rapidly and has been identified as the cause of various chronic diseases, such as cardiovascular disease, cancer, noninsulin-dependent diabetes, and metabolic syndromes [3,4]. Both surgical approaches and non-surgical approaches, including behavior/lifestyle modification and pharmacotherapy, are recommended as treatment options for obesity [5]. In terms of weight loss, the surgical approach is more effective than non-surgical approaches, and it is also more cost effective [6]. However, surgery is associated with adverse effects and long-term follow up is needed [1,7] Pharmacotherapy may be a good alternative for patients who cannot have surgery. Pharmacotherapy may help patients who find it difficult to begin physical activity and modify their lifestyle [8]. Orlistat and sibutramine are the most commonly used agents. Although these drugs have proven their effectiveness, they are known to cause various side effects, such as dry mouth, loss of appetite, insomnia, increased blood pressure and pulse rate, and gastrointestinal disorder [9,10]. For this reason, there is increasing interest in stable obesity treatments using natural products.

*Acanthopanax sessiliflorus* belongs to the family Araliaceae and is widely distributed in Korea, China, and Japan [11]. The fruit of *A. sessiliflorus* has been used as a traditional medicine for hypertension, dizziness, and menopausal women’s syndrome [12], and numerous biological activities have been reported: anti-inflammatory [13], antihypertensive [14], antitumor, and immunostimulating activities [15]. *A. sessiliflorus* fruit can not only be eaten as a medicinal material but also used as a raw material for wine or tea, and it has been registered as a food by the Ministry of Food and Drug Safety [16]. We attempted to confirm that the fruit of *A. sessiliflorus*, which has been identified as a food ingredient, exhibits an anti-obesity effect and examine the metabolic changes in mouse livers according to this effect. In the process of oxidation of fatty acids in the liver, free radicals are generated, oxidative stress may increase, and inflammatory reactions may increase. In addition, obesity and liver damage are closely related. It was reported that liver and hepatocyte are affected by obesity and adipocyte. Chronic lipid accumulation beyond metabolic capacity is known to cause liver cell damage. In addition, obesity is recognized as a cofactor of liver damage induced by chronic hepatitis [17]. Thus, the liver is an organ that can be affected by a high-fat diet or obesity [18], and thus was selected as the target sample in this study.

Metabolomics is considered to be the comprehensive analysis of all metabolites. Previously, metabolomics has been applied and utilized as a functional tool for investigating metabolites [19]. Metabolites are the end products of metabolism, and, because they are the closest to the phenotype, the analysis of these metabolites provides crucial information for understanding various cellular processes [20]. Nuclear magnetic resonance (NMR) spectroscopy not only is used for structural analysis but is also a powerful tool for metabolomics research due to its excellent reproducibility [21].

In this study, the metabolic study of the liver tissue of a high-fat induced mouse model and *A. sessiliflorus* fruit-fed model was conducted using NMR spectroscopy. Both anti-obesity efficacy and biochemical changes characteristic of *A. sessiliflorus* fruit were explored from a metabolomics perspective in our experiment. The results of this paper provide further evidence to understand the mechanism of the anti-obesity effect of *A. sessiliflorus* fruit extracts.

## 2. Results

**Figure 1:**
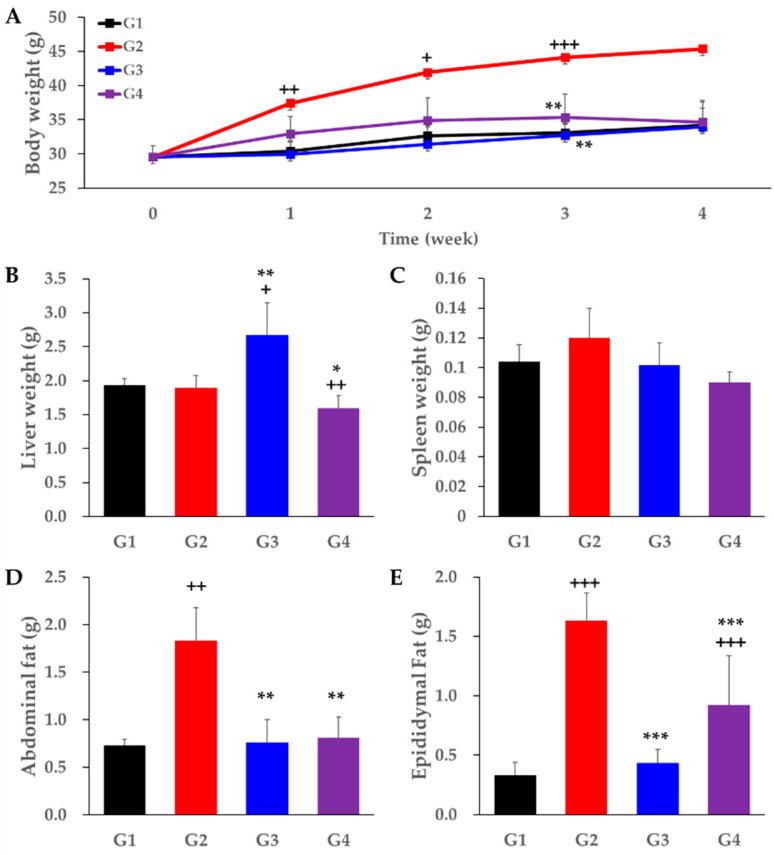
Effect of *Acanthopanax sessiliflorus* fruits extract on body weight and organ weight: (**A**) body weight; (**B**) liver weight; (**C**) spleen weight; (**D**) abdominal fat; (**E**) epididymal fat. Statistically different from the normal diet group (G1) shown with + *p* < 0.05, ++ *p* < 0.01, +++ *p* < 0.001. Statistically different from the high-fat diet group (G2) shown with * *p* < 0.05; ** *p* < 0.01; *** *p* < 0.001.

To confirm the anti-obesity effect of *A. sessiliflorus* fruit, an experiment was performed using high-fat diet (HFD)-induced mice. Animal experiments were conducted by dividing the models into four groups: ICR male mice with normal diet (ND) were the control group (G1), HFD-induced mice were a negative control group (G2), and HFD-induced mice were treated with fenofibrate (G3) and *A. sessiliflorus* fruit extract (G4). As a result of the experiment, the body weight of mice significantly increased in the HFD group (G2) compared to the normal diet group (G1). By comparison, in G3 (*p* < 0.01) and G4 (*p* < 0.01), the body weight of the mice was significantly reduced compared to HFD (G2). The body weight of G3 and G4 was similar to that of G1, which was fed a normal diet (Figure 1A).

The weights of the liver, spleen, epididymal fat, and abdominal fat were measured as obesity parameters caused by administration of *A. sessiliflorus* fruit extract and HFD (Figure 1B–E). After HFD administration, the weight of the spleen increased, and the weight of abdominal fat (*p* < 0.01) and epididymal fat (*p* < 0.001) also significantly increased compared to G1. The weight of the spleen decreased in both G3 and G4 compared to G2. The weight of abdominal fat and epididymal fat significantly decreased in G3 compared to G2, with values of *p* < 0.01 and *p* < 0.001, respectively. In the *A. sessiliflorus* fruit-fed group (G4), the weight of the liver (*p* < 0.05), abdominal fat (*p* < 0.01), and epididymal fat (*p* < 0.001) significantly decreased compared with G2.

**Figure 2:**
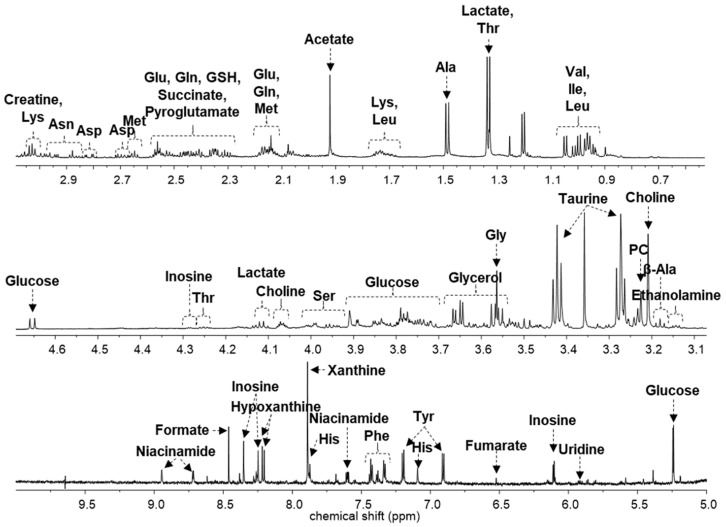
Representative ^1^H nuclear magnetic resonance (NMR) spectrum of mouse liver tissue. The major metabolites are annotated on the spectrum.

**Figure 3:**
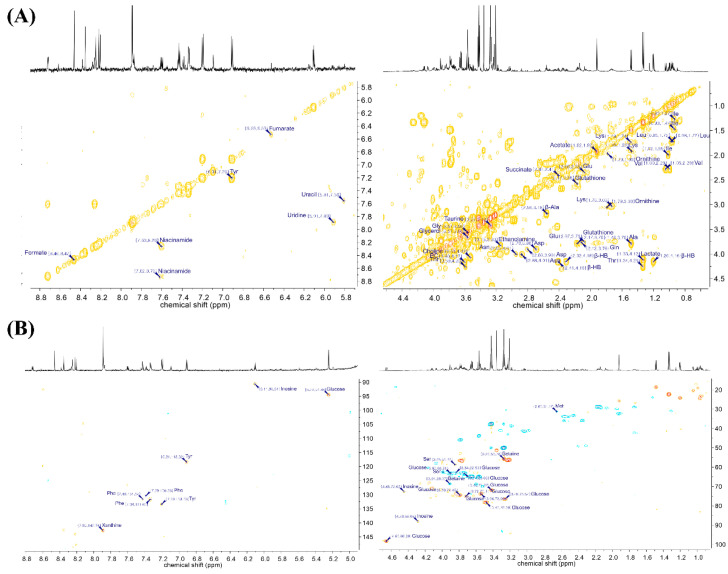
Two-dimensional NMR spectra with annotation of major metabolites of liver sample: (**A**) ^1^H–^1^H correlation spectroscopy (COSY) spectra; (**B**) ^1^H–^13^C heteronuclear single quantum coherence spectroscopy (HSQC)-DEPT NMR spectra.

Liver tissues were analyzed using NMR-based metabolomics to profile metabolites related to the anti-obesity effects of *A. sessiliflorus* fruit on mice. Figure 2 shows the representative NMR spectrum of mouse liver extracts with the annotation of major metabolites. In total, 44 metabolites were identified and quantified in mouse liver extract using the Chenomx 700 MHz metabolite database and 2D NMR data (Figure 3). Their chemical shifts for identification and the concentration data are shown in Table 1. 

**Figure 4:**
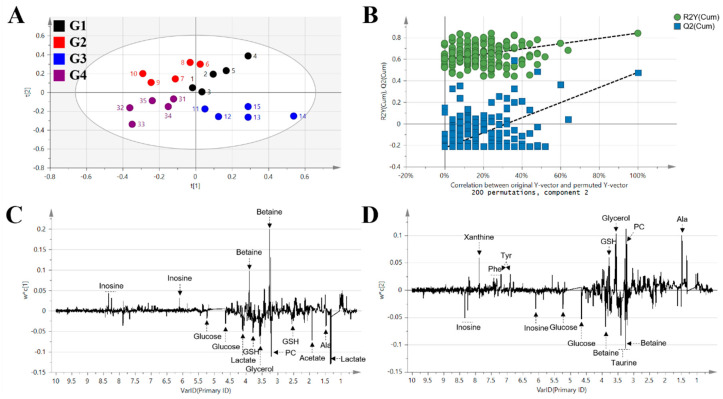
Multivariate statistical analysis of mice liver tissue: (**A**) PLS-DA score plot of all group comparison (R^2^X = 0.481, R^2^Y = 0.504, Q^2^ = 0.268). (**B**) Permutation test of 200 times. Y intercept of R^2^ = 0.595, Y intercept of Q^2^ = −0.225). (**C**) PLS-DA loading plot for the t[1] axis. (**D**) PLS-DA loading plot for the t[2] axis.

Multivariate statistical analysis of the NMR spectra was applied to discriminate the groups. Principal component analysis (PCA) was conducted to determine the unsupervised distribution of samples and for outlier detection. In the PCA score plot, there was no outlier in the samples, and the clustering patterns could not clearly distinguish the groups (data not shown). Therefore, partial least squares discriminant analysis (PLS-DA) was additionally performed (R^2^X = 0.481, R^2^Y = 0.504, Q^2^ = 0.268) (Figure 4A). The model was validated with a permutation test of 200 times. PLS-DA model was not overfitted with Y intercept of R^2^ and Q^2^ less than the original data and Y intercept of Q^2^ less than 0.05 [22,23]. The PLS-DA score plot showed distinct clustering among four groups, and G1 and G2 were clearly distinguished. In particular, it was shown that G2 was separated from G3 and G4 along PLS Components 1 and 2, respectively. The loading plots of Components 1 and 2 are shown in Figure 4C,D, respectively. 

**Figure 5:**
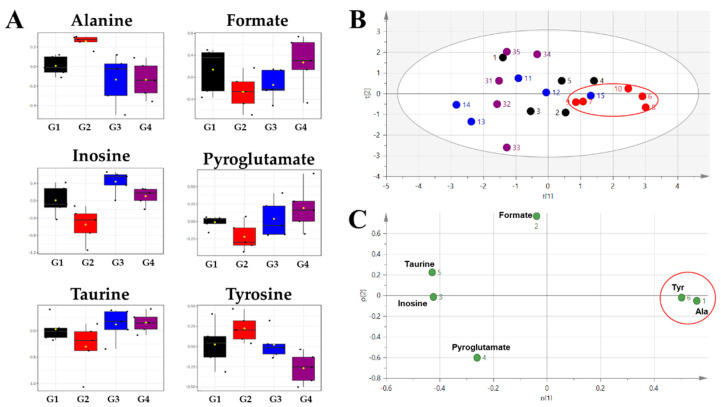
Significantly changed after administration of *A. sessiliflorus* fruits. (**A**) Box plots of selected six metabolites from biomarker analysis. (**B**) PCA score plot of multivariate statistical analysis of selected six metabolites from biomarker analysis. (**C**) PCA loading scatter plot.

Biomarker analysis was performed to select metabolites that were significantly changed by HFD and *A. sessiliflorus* fruit administration. Therefore, comparisons of G1 with G2 to identify the effects of HFD and G2 with G4 to identify the effects of *A. sessiliflorus* fruit administration were conducted in the biomarker analysis. In the results of biomarker analysis, the area under the curve (AUC) of the metabolite was calculated from the receiver operating characteristic (ROC) curve. An AUC value below 0.7 is considered poor, 0.7–0.8 is moderate, 0.8–0.9 is good, and 0.9–1.0 is excellent [24]. In these results, alanine, inosine, formate, pyroglutamate, taurine, and tyrosine had AUC values of 0.7 or more, and these metabolites showed the opposite values of fold change in the comparison of ND/HFD and HFD/HFD + *A. sessiliflorus* fruit (Table 2). Figure 5A shows the box plots of these metabolites for comparison of all groups. The levels of alanine and tyrosine were significantly increased after the high-fat diet. In contrast, the levels of formate, inosine, pyroglutamate, and taurine were identified to decrease significantly after the high-fat diet. Changes in metabolite levels due to the high-fat diet showed a tendency to recover to a similar level to those of the normal diet after administration of fenofibrate and *A. sessiliflorus* fruit extract. We additionally performed a PCA analysis using these selected six metabolites. In the PCA score plot (Figure 5B), G2 was clustered and separated positive region of the t[1] axis. It can be seen that alanine and tyrosine having high concentrations in G2 were represented in the same positive region of the t[1] axis in the PCA loading scatter plot (Figure 5C).

## 3. Discussion

In this study, we confirmed the anti-obesity effect of *A. sessiliflorus* fruits on mouse models given a high-fat diet. The high-fat diet-induced model showed a significant gain in weight compared to the normal diet group, whereas, in the positive control group and the *A. sessiliflorus* fruit treatment group, the body weight of the mouse model was similar to that of the normal diet group. In our study, fenofibrate was used as a positive control. Previous studies have shown that fenofibrate not only inhibits adipocyte hypertrophy [25] but also prevents body weight gain mainly through liver metabolism [26]. It is also known to inhibit visceral obesity and nonalcoholic steatohepatitis [27]. The liver weight of mice did not increase significantly in the high-fat diet group compared to the normal diet group, but it decreased significantly in the *A. sessiliflorus* fruit administration group. By comparison, the positive control group was shown to have increased liver weight, which appeared to be due to lipid deposition in the process of lipid metabolism induced by the high-fat diet [28]. The epididymal fat is used as an appropriate indicator to evaluate changes in white adipose tissue because it is not only sensitive to insulin but also secretes several adipokines [29]. In this experiment, epididymis weight in the high-fat diet group was significantly increased compared to the normal diet group, whereas epididymis weight significantly decreased in the *A. sessiliflorus* fruit administration group. The weights of the spleen and abdominal fat of the *A. sessiliflorus* fruit administration group were similar to those of the normal diet and significantly decreased compared to those of the high-fat diet. From these results, it can be seen that the administration of *A. sessiliflorus* fruits plays a positive role in body and organ weight changes caused by a high-fat diet.

In addition to these results, we analyzed the anti-obesity efficacy of *A. sessiliflorus* fruits from the metabolomics perspective. Mouse livers were analyzed using NMR-based metabolomics. The liver extract contains a lot of high molecular compounds that broaden the signal, so a Carr–Purcell–Meiboom–Gill (CPMG) pulse sequence was used. Since the high molecular compound has a relatively long spin–spin relaxation time (T2), only signals of low molecular weight metabolites can be obtained by the CPMG pulse sequence. We calculated a 90° pulse-width (pw90) for the CPMG pulse sequence and applied 11.82 μs. Acquired NMR spectra were binned, and the binning results were analyzed by multivariate statistical analysis. According to the results of multivariate statistical analysis, the PLS-DA score plot showed a tendency to cluster the high-fat diet group distinctly from the *A. sessiliflorus* fruit and fenofibrate groups. The *A. sessiliflorus* fruit administration group and the fenofibrate group were distinguished from the high-fat diet, but their patterns were slightly different. Unlike metabolic changes formed by a single component’s target mechanism, such as fenofibrate (positive control), the multiple components contained in a mixture such as an extract act in various ways on metabolic changes.

Metabolites were identified and quantified in the spectrum of the liver extract. Metabolite database and 2D NMR spectra were used for the identification of metabolites in the liver extract. The overlapping metabolites were confirmed through the COSY experiment, and the metabolites that were difficult to confirm in the COSY experiment were confirmed by the HSQC-DEPT experiment.

**Figure 6:**
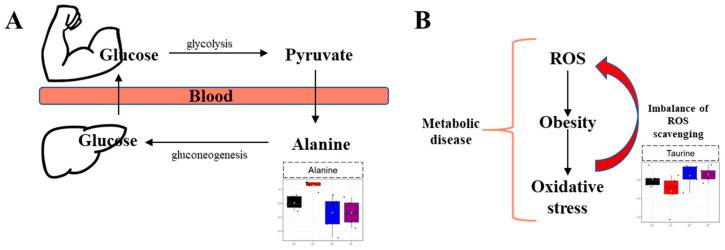
Metabolic pathway affected by *A. sessiliflorus* fruit administration: (**A**) Cahill cycle; (**B**) antioxidant effect. The black dots are the values of the sample represented by the calculated quartiles in each group.

Quantified metabolites were analyzed using biomarker analysis. The result of biomarker analysis is expressed as the ROC curve of each metabolite, and the prediction ability is scored by the AUC value. The ROC curve is drawn with the false positive rate (*x*-axis) and the true positive rate (*y*-axis), and the most ideal cut-off value to distinguish the two groups can be confirmed. In the results of biomarker analysis, metabolites indicating recovery from obesity to normal were identified. Alanine showed a good prediction value of 1.00 in the AUC value of the normal diet/high-fat diet and high-fat diet/high-fat diet + *A. sessiliflorus* fruit comparisons. The glucose–alanine cycle, known as the Cahill cycle, causes alanine to regenerate into glucose in the liver through a series of reactions [30]. In the boxplot of biomarkers, the high-fat diet group showed high levels of alanine concentrations (Figure 6A). According to the study of Song and co-workers, an excessive high-fat diet stimulates alanine gluconeogenesis [31].

In this experiment, the level of formate was decreased in the high-fat diet. These results were the same as those of previous studies, which also showed that the formate level was significantly decreased in the obese group compared to the healthy control group [32]. Inosine, an endogenous metabolic derivative of adenosine, decreased in the high-fat diet group and increased in the positive control group and the *A. sessiliflorus* fruit administration group. This may be due to its cytoprotective effects, as shown in previous studies [33]. This cytoprotective effect of inosine is closely related to antioxidant activity [34,35]. It is also known that inosine has immunomodulatory and neuroprotective effects [36].

Tyrosine is one of the six markers found in our study. The effect of tyrosine, a neutral amino acid, in an animal models of diet-induced obesity has been reported in previous studies. In the previous study, tyrosine administration showed a decrease in liver fatty degeneration and a reduction in ALT, and no distinct fatty degeneration was observed in the liver tissue [37]. In another experiment, fatty degeneration was significantly attenuated in the liver tissue of the tyrosine-treated group, and triglycerides and LDL were normalized in the tyrosine-treated group [38]. These results appear to be due to the modulation of dopamine metabolism by tyrosine.

Taurine was identified to have relatively high content in the *A. sessiliflorus* fruit administration group in the S-line plot, and it was also identified to be one of six specific metabolites in biomarker analysis. Previous studies show that obesity induces oxidative stress, and an increase in reactive oxygen species (ROS) production is also known to occur due to an imbalance in the ROS scavenging system or increased production of oxidative stress in cells [39,40]. In addition, oxidative stress in the obese condition is associated with metabolic syndrome [41], and it is known that, if obesity persists, the activity of related enzymes may decrease due to depletion of the antioxidant source [42]. Therefore, supplementation of antioxidants is recommended to reduce the risk of obesity and its related complications [43].

In this experiment, taurine concentration was relatively increased when *A. sessiliflorus* fruit was administered, which appears to be due to the antioxidant effect of taurine (Figure 6B). Taurine is known to be a representative antioxidant, and previous studies have shown that iron-induced liver damage is reduced by taurine treatment in the murine model; this appears to be the effect of the sulfur moiety of taurine [44]. In addition, the regulation of GSH/GSSH level by taurine appears to play an important role in the cell membrane defense against oxidative stress.

## 4. Materials and Methods

### 4.1. Extraction of Acanthopanax sessiliflorus Fruits

*Acanthopanax sessiliflorus* fruits were harvested in Jeongseon, Republic of Korea. A voucher specimen (NIHHS1501) was deposited at the Herbarium of the Department of Herbal Crop Research, National Institute of Horticultural and Herbal Science, Rural Development Administration, Eumseong, South Korea. *A. sessiliflorus* fruits were ground and homogenized using a mixer and a ball mill, respectively. *A. sessiliflorus* fruits were extracted under reflux for 6 h using 50% aqueous fermented ethanol at 70 °C for 6 h and extracted again for 3 h under the same conditions. After filtering using a 5 μm filter, the extract was concentrated under reduced pressure to obtain 10–20 brix materials. Concentrated extract was sterilized at 80–90 °C for 1 h and then freeze-dried under reduced pressure (−30 °C, 100 mTorr) for 24 h.

### 4.2. Animal Administration

ICR male mice weighing approximately 27–29 g (7 weeks old) used in the experiment were purchased from the Raonbio (Yongin, Gyeonggi-Do, Korea). We obtained institutional review board approval for this study from the Institutional Animal Care and Use Committee of Konyang University (Approval No. P-18-07-A-01). Mice were housed under a controlled environment (12/12 h light-dark cycle, a temperature of 22 ± 2 °C, and 50 ± 10% humidity) for adaption. After the acclimatization period, mice were randomly divided into four groups (*n* = 5 per group) as follows: (G1) ND, normal diet; (G2) HFD, high-fat diet; (G3) Fenofibrate, HFD + 2 mg of fenofibrate; (G4) *A. sessiliflorus* fruits, HFD + 3 mg of *A. sessiliflorus* fruits. The nutritional content of the high-fat diet, unlike the normal diet, contained 34% fat including soybean oil and lard. G3 was used as a positive control group, and fenofibrate was used at 2 mg/hd. Drug and *A. sessiliflorus* fruits extract were orally administered daily for 4 weeks. The body weights of mice were measured every week.

### 4.3. Sample Preparation

Polar metabolites in the liver samples were extracted using a solvent of methanol/water/chloroform. An extraction protocol using the Bligh and Dyer method [45] was optimized in this experiment. After the centrifugation, an aqueous layer of the extract was lyophilized for elimination of the solvent. To dissolve the polar metabolites for the NMR analysis, 560 μL of deuterated sodium phosphate buffer containing 2.000 mM of 3-(trimethylsilyl)-propionic-2,2,3,3-*d_4_* (TSP-*d_4_*) was used. TSP-*d_4_* was used for calibration of the chemical shift (δ 0.00) and quantification of metabolites. For the NMR measurement, samples were transferred to a 5 mm NMR tube.

### 4.4. NMR Data Acquisition and Data Processing

Liver extract samples were measured using a Bruker Avance 700 spectrometer (Bruker Biospin, Rheinstetten, Germany) with a cryogenic triple-resonance probe at a frequency of 700.40 MHz for ^1^H and a temperature of 298 K. One-dimensional (1D) ^1^H-NMR spectra were recorded with a pulse sequence of Carr–Purcell–Meiboom–Gill (Bruker; cpmgpr1d) for suppression of high molecular weight metabolites and water signals, 64 scans, relaxation delay 2 s, and acquisition time 1.802 s. The data of two-dimensional (2D) ^1^H–^1^H correlation spectroscopy (COSY), with 320 × 2048 complex points, spectral width of 12 ppm, 9 dummy scans, and 32 scans, and ^1^H–^13^C heteronuclear single quantum coherence spectroscopy (HSQC-DEPT), with 320 × 1024 complex points, spectral width of 165 ppm for ^13^C (F1) and 12 ppm for ^1^H (F2), 32 dummy scans, and 64 scans, were acquired to confirm the identification of metabolites. The phase and baseline of the NMR spectra were manually corrected with TOPSPIN (4.1.0; Bruker Biospin, Rheinstetten, Germany).

### 4.5. Data Analysis

Metabolites were identified and quantified using Chenomx NMR Suite 8.4 Professional (Chenomx Inc, Edmonton, AB, Canada) with the metabolite library database and 2D data. A relative quantification of metabolites was performed on data normalized according to the TSP signal. Biomarker analysis of quantified metabolic profile was conducted using MetaboAnalyst 5.0 (https://www.metaboanalyst.ca, accessed on 27 June 2021) to evaluate meaningful metabolites. In the results of biomarker analysis, ROC curves of each metabolite were plotted with true positive rate and false positive rate. AUC values, a sorting-based algorithm, were used to measure the predictive abilities. All spectra were binned using Chenomx NMR Suite 8.4 Professional for the multivariate statistical analyses. The binning area of the spectra was from 0.5 to 10 ppm with a binning size of 0.001 ppm. Residual solvent signals of water (4.65–5.1 ppm), ethanol (1.05–1.3 and 3.62–3.67 ppm), and methanol (3.32–3.37 ppm) were excluded and then normalization was performed for the total area. Binning data were aligned using the icoshift algorithm of MATLAB (The MathWorks, Natick, MA, USA). Processed binning results were analyzed with SIMCA 15.0.2 software (Umetrics, Umeå, Sweden). Before the analysis, data were scaled to Pareto scaling. Principal component analysis (PCA) was conducted to show the distribution of unsupervised samples. Partial least squares discriminant analysis (PLS-DA) was performed to show the group clustering.

## 5. Conclusions

This study was conducted to investigate the anti-obesity effect of *A. sessiliflorus* fruits using nuclear magnetic resonance (NMR) spectroscopy-based metabonomics. The PLS-DA score plot showed the separation of the group that was administered *A. sessiliflorus* fruit from the HFD-induced group. As a result of biomarker analysis, six metabolites were identified using the AUC of the metabolites. The six metabolites, including alanine and taurine, may be useful as biomarkers of the anti-obesity effect of *A. sessiliflorus* fruits. The levels of these metabolites were distinguished from the high-fat diet model.

## Figures and Tables

**Table 1:** Identified and quantified metabolites in serum sample from ^1^H−NMR spectra. Values are means (%) ± standard deviations of relative concentrations. A relative quantification of metabolites was performed on data normalized according to the TSP signal.

Compound	Chemical Shifts (Multiplicities) (ppm)	G1 (%)	G2 (%)	G3 (%)	G4 (%)
Acetate	1.91 (s)	2.371 ± 0.212	2.740 ± 0.466	2.266 ± 0.529	2.950 ± 0.665 *
Alanine	1.47 (d), 3.77 (q)	5.013 ± 0.463	5.718 ± 0.576 **	4.421 ± 0.692 ^#^	4.287 ± 0.352 ^##^
Asparagine	2.86 (dd), 2.94 (dd)	1.150 ± 0.305	1.338 ± 0.254	1.395 ± 0.386	1.100 ± 0.236
Aspartate	2.68 (dd), 2.80 (dd)	1.860 ± 0.369	1.736 ± 0.118	1.575 ± 0.107	1.204 ± 0.158 **^,##^
Betaine	3.26 (s), 3.89 (s)	2.466 ± 0.809	1.418 ± 0.327 *	3.591 ± 1.965 ^#^	1.413 ± 0.293 *
β-HB	1.19 (d), 2.29 (dd), 2.40 (dd), 4.14 (m)	0.967 ± 0.316	0.872 ± 0.281	1.794 ± 0.278 **^,##^	1.301 ± 0.470
Choline	3.19 (s), 3.51 (dd), 4.05 (ddd)	1.976 ± 0.489	1.883 ± 0.605	1.837 ± 0.305	2.780 ± 0.239 *^,#^
Creatine	3.02 (s), 3.92 (s)	0.260 ± 0.026	0.252 ± 0.034	0.178 ± 0.042	0.218 ± 0.049
Ethanolamine	3.13 (m), 3.82 (m)	0.633 ± 0.158	0.699 ± 0.070	0.805 ± 0.097 *^,#^	0.847 ± 0.180 *^,#^
Formate	8.44 (s)	1.499 ± 0.507	1.009 ± 0.326	1.069 ± 0.326	1.609 ± 0.651
Fumarate	6.51 (s)	0.045 ± 0.012	0.059 ± 0.013	0.017 ± 0.007 **^,###^	0.053 ± 0.012
Glucose	3.24 (m), 3.40−3.48 (m), 3.53 (dd), 3.70−3.89 (m), 4.64 (d), 5.23 (d)	6.133 ± 1.564	9.853 ± 2.912	10.228 ± 4.246	10.837 ± 4.365 *
Glutamate	2.05(m), 2.12 (m), 2.32−2.35 (m)	3.835 ± 0.579	3.744 ± 0.367	3.542 ± 0.446	3.424 ± 0.159
Glutamine	2.12−2.13 (m), 2.42−2.46 (m)	2.854 ± 0.253	2.749 ± 0.228	3.721 ± 0.653 *^,#^	2.543 ± 0.163
Glutathione	2.14−2.16 (m), 2.50−2.56 (m)	1.528 ± 0.207	2.078 ± 0.553	1.509 ± 0.238	1.836 ± 0.277 *
Glycerol	3.55 (dd), 3.64 (dd), 3.77 (m)	8.292 ± 0.613	8.232 ± 0.895	5.625 ± 0.956 *^,#^	7.515 ± 1.411
Glycine	3.55 (s)	5.878 ± 0.512	6.260 ± 0.332	5.107 ± 0.109 *^,#^	5.682 ± 0.187 *
Histidine	7.08 (s), 7.86 (s)	0.605 ± 0.055	0.569 ± 0.077	0.681 ± 0.094 **^,##^	0.458 ± 0.038 **
Hypoxanthine	8.19 (s), 8.20 (s)	1.362 ± 0.202	1.172 ± 0.440	1.064 ± 0.337	1.215 ± 0.425
Inosine	4.27 (m), 4.43 (dd), 6.09 (d), 8.23 (s), 8.34 (s)	0.830 ± 0.252	0.473 ± 0.194 *	1.220 ± 0.280 ^##^	0.828 ± 0.156 ^#^
Isoleucine	0.93 (t), 1.00 (d), 1.25 (m), 1.46 (m), 1.97 (m), 3.66 (d)	1.048 ± 0.162	1.081 ± 0.130	1.214 ± 0.135 *^,##^	0.888 ± 0.067
Lactate	1.32 (d), 4.10 (q)	5.074 ± 1.099	6.627 ± 0.609	5.000 ± 0.935	6.862 ± 0.994 *
Leucine	0.94 (d), 0.96 (d), 1.67−1.74 (m)	2.445 ± 0.605	2.294 ± 0.424	2.723 ± 0.633 ^#^	1.798 ± 0.192
Lysine	1.44−1.50 (m), 1.72 (m), 1.88−1.92 (m), 3.02 (t)	1.869 ± 0.297	1.848 ± 0.396	1.896 ± 0.373	1.573 ± 0.213
Mannose	3.93−3.94 (m), 5.18 (d)	0.624 ± 0.142	0.615 ± 0.094	0.536 ± 0.127	0.332 ± 0.053 **^,###^
Methionine	2.11−2.19 (m), 2.63 (t)	0.930 ± 0.179	0.966 ± 0.186	0.939 ± 0.175	0.706 ± 0.088 ^#^
Niacinamide	7.59 (dd), 8.24 (dd), 8.70 (dd), 8.93 (s)	0.935 ± 0.110	0.847 ± 0.126	0.836 ± 0.088	0.851 ± 0.054
PC	3.21 (s), 3.58 (m), 4.15 (m)	0.836 ± 0.179	0.818 ± 0.137	0.515 ± 0.101 *^,#^	0.837 ± 0.157
Ornithine	1.74 (m), 1.82 (m), 1.93 (m), 3.04 (t)	0.976 ± 0.276	0.959 ± 0.184	0.890 ± 0.111	0.602 ± 0.094 *^,##^
Phenylalanine	3.12 (dd), 7.32 (m), 7.36 (m), 7.42 (m)	0.835 ± 0.225	0.879 ± 0.229	0.796 ± 0.127	0.605 ± 0.080 ^#^
Pyroglutamate	2.02 (m), 2.38−2.41 (m), 2.50 (m), 4.17 (dd)	0.774 ± 0.088	0.678 ± 0.049	0.754 ± 0.111	0.816 ± 0.162
Serine	3.83 (dd), 3.94 (dd), 3.98 (dd)	3.064 ± 0.679	3.170 ± 0.360	2.780 ± 0.483	1.859 ± 0.297 **^,##^
Succinate	2.39 (s)	0.111 ± 0.065	0.106 ± 0.040	0.172 ± 0.054 ^#^	0.131 ± 0.024
Taurine	3.26 (t), 3.41 (t)	20.324 ± 2.413	16.197 ± 4.497	20.811 ± 3.911	20.823 ± 3.400
Threonine	1.32 (d), 3.58 (d), 4.24 (m)	1.760 ± 0.291	1.746 ± 0.240	1.523 ± 0.234	1.321 ± 0.100 *^,#^
TMAO	3.26 (s)	0.369 ± 0.059	0.063 ± 0.018 ***	0.051 ± 0.014 ***	0.059 ± 0.015 ***
Tryptophan	7.27 (t), 7.32 (s), 7.53 (d), 7.72 (d)	0.107 ± 0.042	0.117 ± 0.020	0.117 ± 0.022	0.121 ± 0.015
Tyrosine	3.05 (dd), 3.94 (dd), 6.89 (m), 7.18 (m)	1.048 ± 0.214	1.175 ± 0.194	0.979 ± 0.169	0.798 ± 0.081 ^##^
Uracil	5.79 (d), 7.54 (d)	0.173 ± 0.083	0.121 ± 0.024	0.108 ± 0.030	0.098 ± 0.028
Uridine	5.89 (d), 5.91 (d), 7.87 (d)	0.068 ± 0.026	0.053 ± 0.018	0.078 ± 0.019 ^#^	0.068 ± 0.011
Valine	0.98 (d), 1.03 (d), 2.26 (m), 3.60 (d)	1.929 ± 0.368	1.807 ± 0.238	1.848 ± 0.278	1.519 ± 0.161
Xanthine	7.87 (s)	2.583 ± 0.470	2.472 ± 0.236	1.706 ± 0.315 *^,#^	2.431 ± 0.561
myo-Inositol	3.27 (t), 3.53 (dd), 3.61 (t), 4.06 (t)	0.900 ± 0.122	0.941 ± 0.150	0.648 ± 0.252	1.016 ± 0.159
β-Alanine	2.55 (t), 3.17 (t)	1.661 ± 0.336	1.567 ± 0.136	1.435 ± 0.289	1.785 ± 0.180 ^##^

^#^ Significantly different to G2 with *p*-value < 0.05; ^##^ significantly different to G2 with *p*-value < 0.01, ^###^ significantly different to G2 with *p*-value < 0.001. * Significantly different to G1 with *p*-value < 0.05; ** significantly different to G1 with *p*-value < 0.01; *** significantly different to G1 with *p*-value < 0.001.

**Table 2:** Area under the curve (AUC) values of metabolites over 0.7 obtained from biomarker analysis.

Compounds	ND/HFD			HFD/HFD + *A. sessiliflorus* Fruit		
	AUC	*t*-tests	Log2FC	AUC	*t*-tests	Log2FC
Alanine	1.00	0.0014	−0.18989	1.00	0.0044	0.41554
Inosine	0.88	0.0457	0.81044	0.96	0.0137	−0.80721
Formate	0.80	0.1264	0.57076	0.84	0.0722	−0.67269
Pyroglutamate	0.76	0.0711	0.19067	0.88	0.0642	−0.26675
Taurine	0.76	0.1923	0.32743	0.92	0.0689	−0.36244
Tyrosine	0.72	0.2019	−0.16551	1.00	0.0050	0.55755

## Data Availability

Data is contained within the article.
